# Identification of a Novel Human Rhinovirus C Type by Antibody Capture VIDISCA-454

**DOI:** 10.3390/v7010239

**Published:** 2015-01-19

**Authors:** Seyed Mohammad Jazaeri Farsani, Bas B. Oude Munnink, Marta Canuti, Martin Deijs, Matthew Cotten, Maarten F. Jebbink, Joost Verhoeven, Paul Kellam, Katherine Loens, Herman Goossens, Margareta Ieven, Lia van der Hoek

**Affiliations:** 1 Laboratory of Experimental Virology, Department of Medical Microbiology, Center for Infection and Immunity Amsterdam (CINIMA), Academic Medical Center of the University of Amsterdam, Amsterdam 1105 AZ, the Netherlands; E-Mails: s.m.jazaerifarsani@amc.uva.nl (S.M.J.F.); b.b.oudemunnink@amc.uva.nl (B.B.O.M.); marta.canuti@gmail.com (M.C.); m.deijs@amc.uva.nl (M.D.); m.f.jebbink@amc.uva.nl (M.F.J.); verhoevenjtp@googlemail.com (J.V.); c.m.vanderhoek@amc.uva.nl (L.V.D.H.); 2 Department of Virology, Tehran University of Medical Sciences, Tehran 14176-14411, Iran; 3 Department of Virus Genomics, Wellcome Trust Sanger Institute, Hinxton CB10 1SA, UK; E-Mails: mc13@sanger.ac.uk (M.C.); pk5@sanger.ac.uk (P.K.); 4 Department of Medical Microbiology, Vaccine and Infectious Disease Institute, University Hospital, Antwerp B-2650, Belgium; E-Mails: katherine.loens@uza.be (K.L.); herman.goossens@uza.be (H.G.); greet.ieven@uza.be (M.I.)

**Keywords:** rhinovirus C54, virus discovery, antibody capture VIDISCA-454

## Abstract

Causative agents for more than 30 percent of respiratory infections remain unidentified, suggesting that unknown respiratory pathogens might be involved. In this study, antibody capture VIDISCA-454 (virus discovery cDNA-AFLP combined with Roche 454 high-throughput sequencing) resulted in the discovery of a novel type of rhinovirus C (RV-C). The virus has an RNA genome of at least 7054 nt and carries the characteristics of rhinovirus C species. The gene encoding viral protein 1, which is used for typing, has only 81% nucleotide sequence identity with the closest known RV-C type, and, therefore, the virus represents the first member of a novel type, named RV-C54.

## 1. Introduction

Viral respiratory infections cause serious health problems in human populations. Many agents can infect and replicate in the respiratory tract, however, causative agents for 30 percent of respiratory infections remain unidentified [1,2,3], suggesting that not-yet-identified pathogens, including viruses, remain to be discovered. Many of these unknown viruses may be difficult to propagate in culture and for this reason, culture- and sequence-independent metagenomic methods are needed to identify these agents. VIDISCA-454 (virus discovery cDNA-AFLP, amplified fragment-length polymorphism combined with Roche 454 high-throughput sequencing) includes a reverse transcription, second strand synthesis, restriction enzyme digestion, ligation to adaptors, PCR amplification, and deep sequencing to generate virus sequences independent of the viral characteristics and its genome composition [4,5,6,7,8,9,10]. Discovery of unknown pathogens in respiratory samples can be complicated by high concentrations of ribosomal RNA (rRNA), which competes for sequence space, reducing the number of reads that originate from viruses [11]. Furthermore, attributing the presence of a virus in patient material to a disease, or even an infection, can be a challenge. In particular, new viruses have been identified in human respiratory samples by sensitive deep sequencing techniques, but a clear association of a new virus with a disease requires further detailed investigations (e.g., torque teno mini virus and gamma human papillomavirus are not yet established as human pathogens [6,12,13]). Another difficulty is to recognize new viruses among the massive amount of sequence data generated. With most currently used methods, virus identification is dependent on the identity of the new virus to already known virus genomes. National Center for Biotechnology Information (NCBI) Blast tool searches can identify viruses if a closely related virus is present in GenBank, but highly divergent virus sequences may remain unrecognized.

To overcome these limitations in virus discovery, we recently developed a modification of the VIDISCA-454 method by adding an antibody capture step. This method uses host antibodies elicited by an infection, obtained from convalescent serum one month after the respiratory illness. These antibodies are used to enrich the causative viral agent from the respiratory material that was collected during the acute phase of the infection. The advantage of the antibody capture approach is multi-fold: (1) it decreases the background rRNA increasing the relative percentage of virus sequence reads (2) it identifies viruses that induced an immune response in the patient, which increases the chance to find a pathogenic virus; and (3) it allows the identification of new viruses that have very low identity to known viruses [14]. In this study we identified a new type of rhinovirus C with this novel antibody capture VIDISCA-454 method.

Rhinoviruses are non-enveloped viruses with a single-stranded positive-sense RNA genome of approximately 7200 nt. Their genome encodes a single open reading frame (ORF) encoding a polyprotein that is cleaved into four structural proteins (VP1, VP2, VP3 and VP4) and seven non-structural proteins (2A, 2B, 2C, 3A, 3B, 3C and 3D) [15]. VP1-4 form the capsid that surrounds the RNA genome, while the remaining nonstructural proteins are involved in viral genome replication and virion assembly [16]. Rhinoviruses belong to the *Enterovirus* genus within the *Picornaviridae* family [17] and three species are known: RV-A, RV-B, and RV-C [17]*.* Rhinoviruses were first discovered in the 1950s and have been linked to the majority of upper respiratory tract infections in humans. Furthermore, RV-C infection is found in about half of all rhinovirus infections in young children [18]. Despite being highly prevalent, RV-Cs are however difficult to culture, with replication reported on commercial 3D human upper airway epithelia [19], sinus mucosal organ culture [18], human primary bronchial [20], and sinus epithelial cells [21]. According to the latest classification, 53 Rhinovirus C types have been identified thus far [22] with the novel virus identified here named RV-C54 by the International Committee on Taxonomy of Viruses (ICTV) *Picornaviridae* Study Group [22].

## 2. Materials and Methods

### 2.1. Clinical Samples

The respiratory sample was collected in 2009 during the GRACE study [23,24] from a 35-year-old female patient. A flocked nasopharyngeal swab (Copan, Brescia, Italy) was collected in universal transport medium (UTM). The serum was collected 5 weeks after acute infection (convalescent serum), at that time the patient was symptom free. During the acute phase the patient had respiratory complaints including rhinorrhea, severe shortness of breath, wheeze and phlegm production. The sample tested negative by real time PCR for known viruses including influenza virus A, influenza virus B, respiratory syncytial virus, rhinoviruses, human parainfluenza viruses 1–4, adenovirus, bocavirus, human metapneumovirus, polyomaviruses KI and WU, and human coronaviruses-OC43, -229E, and -NL63. Furthermore, all bacterial diagnostics remained negative including *Mycoplasma pneumoniae*, *Chlamydophila pneumoniae*, *Bordetella pertussis*, *Legionella pneumophila*, *Streptococcus pneumoniae*, and *Haemophilus* spp.

### 2.2. Ethical Approval

The ethics review committee in Barcelona (Spain) Comitè ètic d'investigació clínica Hospital Clínic de Barcelona approved the study.

### 2.3. Antibody Capture

The respiratory sample was centrifuged (10,000 *g*) and 150 µL of the supernatant was mixed with 50 µL of a mixture containing Dynabeads protein A, Dynabeads protein G, and BcMag protein L magnetic beads (suppliers Invitrogen, Carlsbad, CA, USA and Bioclone, San Diego, CA, USA). After 20 min incubation, 10 µL of convalescent serum was added to the mixture. After a subsequent 20 min incubation with continuous shaking at room temperature, samples were washed six times with PBS using a magnetic particle concentrator. Universal transport medium with TURBO™ DNase (Ambion, Austin, TX, USA) was added to the antibody-antigen complex and samples were incubated at 37 °C for 30 min. The complexes were lysed with Boom-lysis buffer L6 [25] and the lysate was used to isolate the nucleic acids with the Boom extraction method with elution in sterile water [25].

### 2.4. VIDISCA and Roche Titanium-454 Sequencing

VIDISCA-454 was performed on the sample (input) and after antibody-capture. The pretreatment to enrich for capsid protected nucleic acids in the input was performed as previously described [9]. In short, input sample was centrifuged for 10 min at 10,000 *g* and the supernatant was treated with TURBO™ DNase (Ambion). Subsequently, nucleic acids were extracted by the Boom extraction method [25], with elution in sterile water containing rRNA-blocking oligonucleotides to prevent amplification of rRNA [9]. The nucleic acids from the input original samples (“input”) and nucleic acids from the captured material (“enriched”) were reverse transcribed into cDNA with Superscript II (Invitrogen) using non-ribosomal random hexamers [26]. Second strand DNA synthesis was performed with Klenow fragment (New England Biolabs, Ipswich, MA, USA) and double-stranded DNA was purified by phenol/chloroform extraction and ethanol precipitation. The double stranded DNA was digested with MseI restriction enzyme (New England Biolabs). Adaptors were ligated to the digested fragments followed by a size-selection purification to reduce the amplification of DNA fragments smaller than 200 bp using Agencourt AMPure XP beads (Beckman Coulter, MA, USA). A 28-cycle PCR with adaptor-binding primers was executed, combined with a purification of the PCR products (Agencourt AMPure XP PCR, Beckman Coulter, MA, USA) to remove excess primers and short PCR-fragments. The DNA concentration was determined with the Quant-it dsDNA HS Qubit kit (Invitrogen) and the KAPA Library Quantification kit (Kapa Biosystems, Wilmington, MA, USA). Subsequently, the Bioanalyser (hsDNA chip, Agilent Technologies, Santa Clara, CA, USA) was used to determine the average nucleotide length of the library which was diluted until 10^6^ copies/µL, clonally amplified in an emulsion PCR according to the suppliers’ protocol (LIB-A SV emPCR kit, Roche, Mannheim, Germany), and sequenced on a GS FLX Titanium PicoTiterPlate (70 × 75) with the GS FLX Titanium XLR 70 Sequencing kit (Roche, Mannheim, Germany). Adaptor sequences and rRNA sequences were trimmed and removed from the obtained sequence reads.

### 2.5. Xcompare2 Pipeline

To identify sequences enriched by antibody capture, reads obtained from the input sample (input dataset) and from the post capture sample (enriched dataset) were compared to each other using the Python (version 2.7.8) based *Xcompare2* pipeline (source available on request). The *Xcompare2* script creates a custom BLAST nucleotide database [27] comprised of all reads within the input dataset, and subsequently identifies identical or near-identical sequences (based on sequence identity) within the same input dataset by performing a stringent BLASTN search (Dust filter disabled, E-value: 3E-60, word size: 11, match/mismatch scores 1/−2, gap existence/extension penalty: 5/2) with this database utilizing the input sequences as a query. These BLAST results are used to construct a new library, containing both unique read sequences and consensus sequences of reads found multiple times (aligned with MUSCLE (Version 3.8.31, 2013) [28,29] with the following settings: maximum number of iterations: 1; diagonal optimization enabled, and metadata on sequence abundance). This library is converted into a second custom BLAST database (flat database) to which sequences from the enriched dataset are compared using BLASTN (Dust filter disabled, E-value: 3E-60, word size: 11, match/mismatch scores 1/−2, gap existence/extension penalty: 5/2). The results obtained from the second stage BLAST analysis are used to identify sequences, which are either unique or more frequently detected in one of the two datasets (input or enriched). For every sequence present in both the input and enriched dataset the Enrichment Index is calculated as the ratio between the percentage of sequence space occupied by each unique sequence in the input dataset (number of reads in the input library matching the unique sequence divided by the total number of reads of the library) and the enriched dataset (number of reads in the enriched library after antibody capturing matching the unique sequence divided by the total number of reads of the library). Sequences which are observed solely in either the input or the enriched datasets are automatically flagged and stored separately for manual inspection. In this study all sequences with an Enrichment Index higher than 1.0 and sequences identified only in the enriched sequence set, were extracted and further analyzed.

### 2.6. Sequence Analysis

Sequences were compared with all available sequences in the non-redundant GenBank database [30] via the BLASTN (http://blast.ncbI.nlm.nih.gov/Blast.cgi) tool [31]. The following settings were used: expect threshold: 1000, Match/ Mismatch Scores: 1/−1, Gap Costs: Existence: 2 Extension: 1. The output was subsequently used to create a taxonomic classification of the reads with Megan software version 5.6.1, (University of Tübingen, Tübingen, Germany, 2014) [32]. The following settings were used: Min Support: 1, Min Score: 10, Top Percent: 100 and Max expected: 100.

### 2.7. Full-Length Genome Sequencing

Full length sequencing of the rhinovirus C genome was performed via the VIDISCA procedure for enrichment of particle-protected nucleic acid isolation and conversion of RNA into double stranded DNA (described above), followed by Illumina MiSeq deep sequencing, exactly as described [33]. The complete genome sequence of the new type of RV-C has been deposited in the GenBank sequence database under accession number KP282614.

### 2.8. Phylogenetic Analysis

Phylogenetic analyses (neighbor-joining method, Maximum Composite Likelihood model) were conducted using MEGA, version 6 [34]. A thousand replicates were performed to add significance to the branches of the tree, and a bootstrap value >80 was considered significant.

### 2.9. Virus Genome Characterization

NetPicoRNA program [35] was used to predict potential cleavage sites of the 2A and 3C protease. Mfold software (State University of New York at Albany, New York, NY, USA, 2003) [36] was applied to generate a viral RNA secondary structure. The identity was investigated by pairwise alignment using ClustalX Version 2.1 (University College Dublin, Dublin, Ireland, 2007) [37]. The recombination detection program (RDP) version 4 (University of Cape Town, Cape Town, South Africa, 2014) [38] was used to identify genetic recombination events.

## 3. Results

Within the GRACE study lower respiratory tract infections are examined for known viruses and bacteria [39]. Some of the infections remain unexplained as all diagnostic tests remain negative. Such a patient, with a rhinorrhea, severe shortness of breath, wheeze and phlegm production was the source of the virus material examined here.

Since convalescent serum was available, antibody capture VIDISCA-454 was performed [14] in order to reduce rRNA background and focus only on viruses to which the patient had developed an immune response. In total 6569 sequence reads were obtained from the untreated material and 11,819 reads from the antibody-mediated captured material. As expected the amount of rRNA in the capture dataset was low (3% in captured sample compared to 35% in the untreated input).

To identify sequences enriched by antibody capture, reads obtained from the input sample (input dataset) and from the post capture sample (enriched dataset) were compared to each other using the X*compare2* pipeline (see Materials and Methods). The script creates and compares two unique fragment libraries for both input and antibody-captured datasets to calculate the Enrichment Indices. Two consensus sequences only found in the captured sequence library showed some identity to rhinovirus C. This enrichment indicates that antibodies from the patient had captured the virus, strengthening the assumption that rhinovirus C was the cause of the lower respiratory tract infection.

The shared nucleotide identity of the VIDISCA sequences with known rhinovirus C sequences was on average 73%. Since only full genome sequences can reveal whether it truly represents a new rhinovirus type or a recombinant virus, the complete genome sequence was determined with the ViSeq method, that uses Illumina MiSeq sequencing and has the capacity to reveal a full-length virus genome sequence within one run [33]. The full-length genome was 7054 nt in size, with a single large ORF of 6447 nt. The base composition of the RNA genome is rich in A (31.5%) and U (25.2%) and relatively poor in G (22.0%) and C (21.3%), a property similar to other rhinoviruses [40]. The single large ORF runs from nucleotide position 608 to 7054 preceded by a 5' untranslated region (UTR). The ORF encodes a polyprotein of 2148 amino acids, which in analogy to other rhinoviruses is probably cleaved by virally encoded proteases (2A and 3C) to yield 11 proteins. Proteinase cleavage sites on the polyprotein were predicted by the NetPicoRNA program [35] and potential cleavage sites are listed in Table 1. At the VP4/VP2 junction there is the Met 67/Ser 68 cleavage site, similar to what has been described for other RV-Cs [41]. Another characteristic typical for RV-C is the isoleucine at the termination of the 3D polymerase gene. Additionally, RV-C54 encodes this isoleucine.

**Figure 1:**
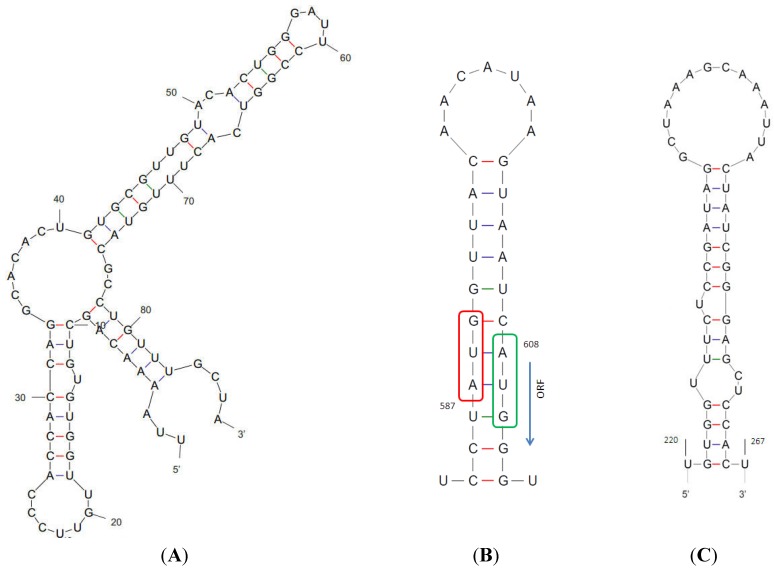
Predicted RNA structures of (**A**) the complete 5' UTR; (**B**) ribosomal entry structure within the IRES; (**C**) cis acting regulatory element in the VP2/VP4 gene.

Analysis of the rhinovirus genome secondary structure by Mfold revealed that the 607 nt long 5' UTR contains a strong secondary structure at position 1 to 87 (Figure 1A), which is somewhat different from the cloverleaf secondary structure motif described previously [40,41], as one arm is missing. This strong secondary structure is followed by a spacer tract (88-GCUAUCCCCCCCAACUUAUGUAAU-107, [42]) and an internal ribosome entry site (IRES). The IRES length is 498 nt (109–606) and forms an unbranched stem in the 3' part of the predicted RNA structure. As has been shown for other rhinoviruses, in the new type of RV-C the last AUG of the IRES and the open reading frame AUG create a variably pair (Figure 1B), facilitating ribosome entry in a specific mechanism known for rhinoviruses [42]. Within the VP4/VP2 gene of RV-Cs, there is a cis-acting regulatory element (CRE), and in the RV-C54 this CRE is located between nucleic acid positions 220–267 (Figure 1C).

**Table 1:** 2A and 3C protease cleavage sites prediction.

Position	Protease	Sequence
841	2A	DSIKTA * GPSDL
330	3C	SNRTQ * GLPV
537	3C	VQSGQ * GAIL
983	3C	LATTQ * GPIT
1329	3C	LVIRQ * GFKT
1407	3C	NAIFQ * GLGS
1482	3C	LCMTQ * GAYT
1504	3C	RAVVQ * GPQH
1687	3C	FVESQ * GEII

**Figure 2:**
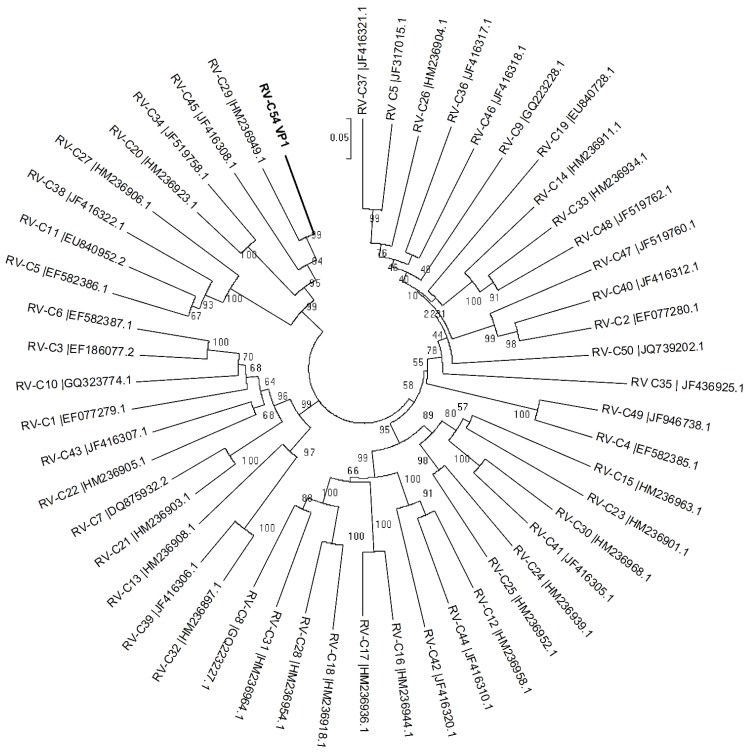
Phylogenetic tree based on nucleotide sequences of the VP1 gene. The neighbor-joining tree (Maximum Composite Likelihood model) was evaluated by 1000 bootstrap pseudo-replicates. RV-C54 is indicated in bold.

**Figure 3:**
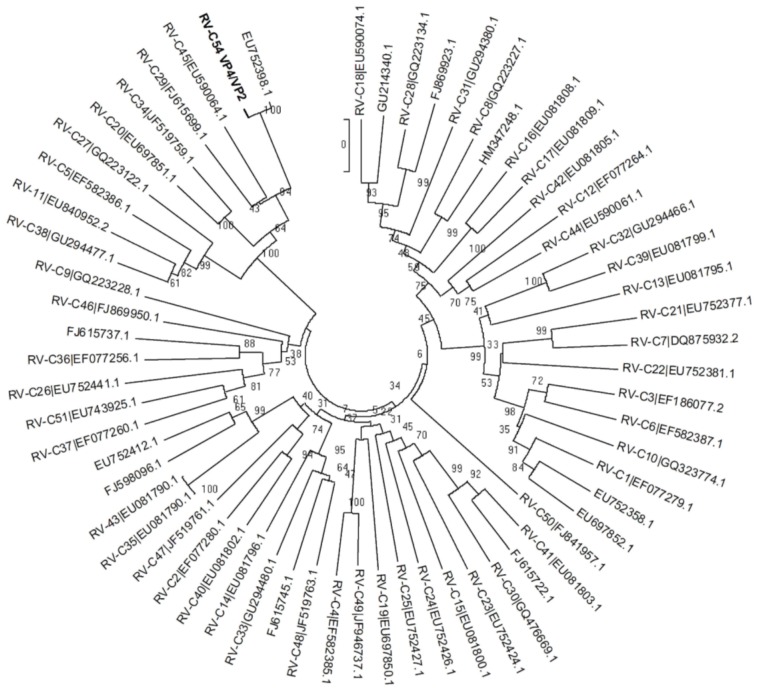
Phylogenetic tree based on nucleotide sequence of the VP4/VP2 gene. The neighbor-joining tree (Maximum Composite Likelihood model) was evaluated by 1000 bootstrap pseudo-replicates. RV-C54 is indicated in bold. Sequences of strains awaiting complete genome or VP1 sequencing, and thus their final type assignment, are indicated with GenBank accession numbers only.

A comparison of the VP1 encoding region revealed that the new virus shares 81% nucleotide identity with its closest relative RV-C29. Phylogenetic analysis was performed based on the VP1 and VP4/VP2 regions using available sequences in the GenBank and the inferred phylogenic trees are shown in Figure 2 and Figure 3. The closest relatives based on the VP4/VP2 sequences of strains that are completely typed are RV-C45 and RV-C29 with 85.0% and 84.8% identity, respectively. However, there are eleven additional strains of which only the VP4/VP2 sequences are available in GenBank. These strains are awaiting type assignments when their complete genome or VP1 sequences have been determined. The VP4/VP2 sequence of RV-C54 has close identity with one of these unassigned strains (95% at nt level: rhinovirus isolate RV1039 (EU752398.1), see Figure 3). Analysis by the ICTV picornavirus study group using the complete viral genome sequence confirmed that our virus is the prototype of a new type, named RV-C54.

**Figure 4:**
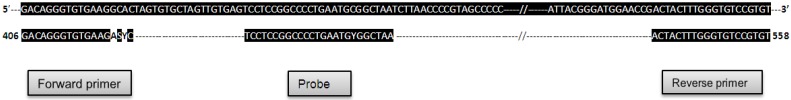
Alignment of primers and probe of the RV diagnostic PCR with the RV-C54 virus genome.

Attempts to culture the virus on differentiated airway epithelial cells (HAE) failed. As the virus was missed in diagnostics, the primers and probe (designed based on 5' UTR region of the RV-C genomes, nucleic acid positions 406–558) sequences of the real time PCR for rhinoviruses were examined in more detail. There are two mismatches in one of the primer binding site used for rhinovirus PCR, which probably caused the negative result in the RV diagnostic test (Figure 4).

## 4. Discussion

In this study a new type of rhinovirus (RV-C54) was identified in a patient with respiratory symptoms. The virus was discovered using antibody capture VIDISCA-454, a culture- and sequence-independent virus discovery technique applicable to detect both RNA and DNA viruses. We have previously shown that the method facilitates virus identification by enriching viral material with convalescent autologous patient antibodies prior to deep sequencing [14]. The requirement that an agent is recognized by the patient’s antibodies adds an important selection for immunogenic viruses [14]. The discovery of a new rhinovirus type by the antibody capture technique demonstrates that the method can be used on clinical samples and that it has good potential to identify novel immunogenic and likely pathogenic viruses. Although the immunogenicity of rhinoviruses is known, in case of a completely new virus for which no prior data is available, the antibody capture gives a strong indication of infectivity in human cells and immunogenicity. The enrichment index can be used to exclude non-immunogenic viruses or viruses from other sources, including ingredients used in sample preparation or contaminants of the sample.

The full genome sequence of the novel virus was used to classify the virus. RV-C strains are not easily cultivable *in vitro*, and RV-C typing, thus, far has been based on phylogenetic analysis with the VP1, VP4/VP2 and the 5' UTR coding region [17]. According to the most recent proposal for classification of rhinoviruses, a threshold of 13% divergence for VP1 nucleotide sequences is used for type assignment of RV-A and RV-C [17], and 12% divergence at the nucleotide level of RV-B VP1 [17]. Since RV-C54 virus has 19% divergence in the VP1 region, the virus is classified as a new type [22].

One of the important causes for diversification of *Rhinovirus* species is genetic recombination [15]. This can create additional serotypes. We compared RV-C54 by RDP software with 30 full-length genome sequences of RV-Cs available in GenBank, but found no evidence of recombination with known RV-Cs. Interspecies recombination has also been described for the 5' UTR and 2A region between RV-A and RV-C, therefore, we checked if there was more identity with RV-A at these parts of the genome, however, there was no evidence for recombination with any of the known RV-A types.

In conclusion, our results highlight the strength of the antibody capture method to identify novel viruses that have elicited an immune response in the host.
